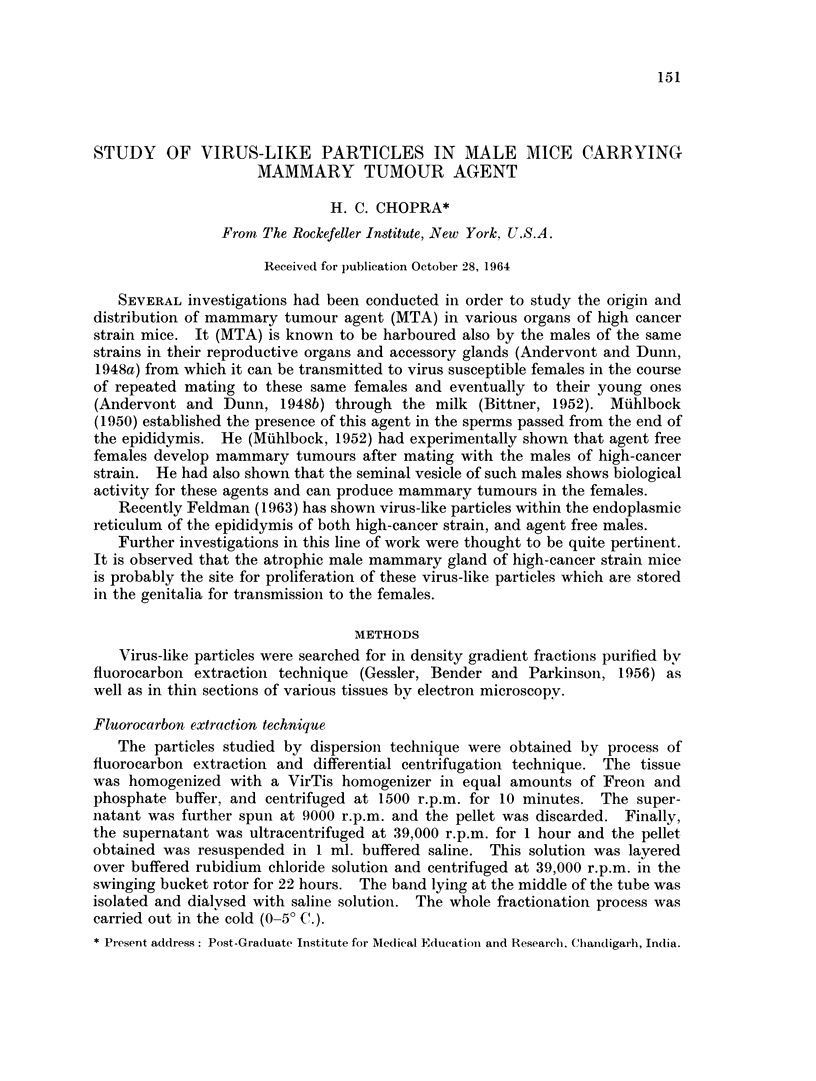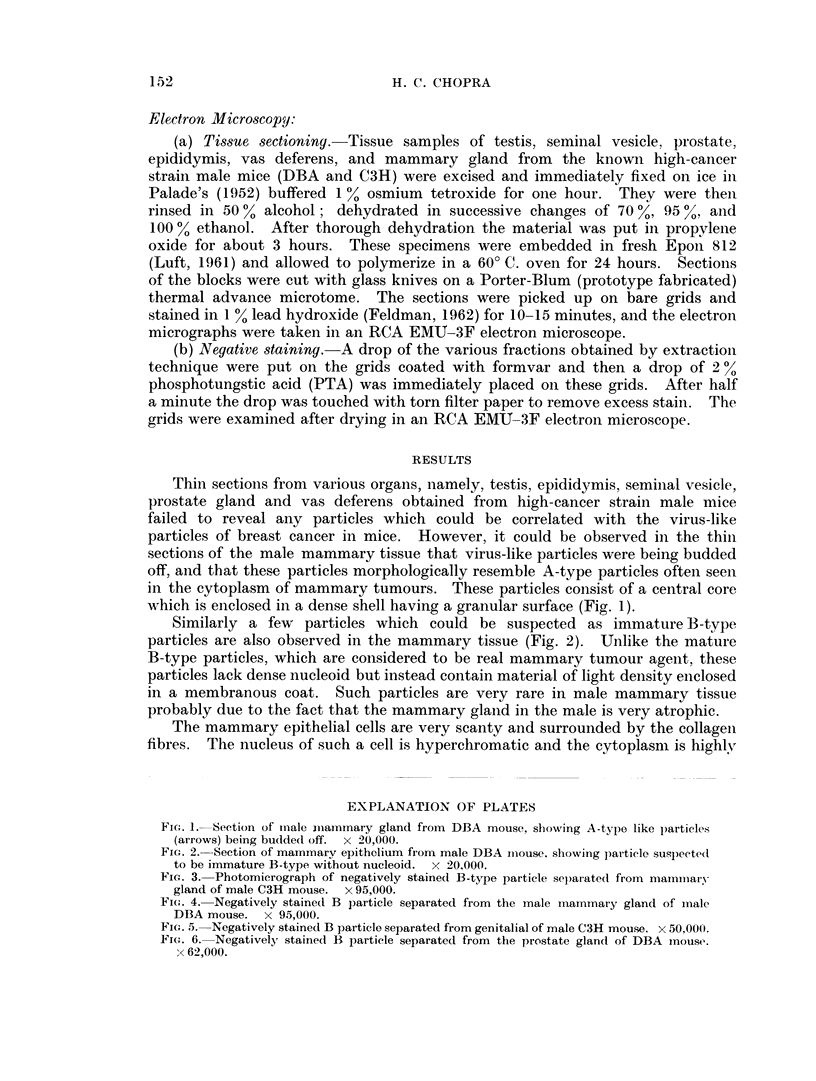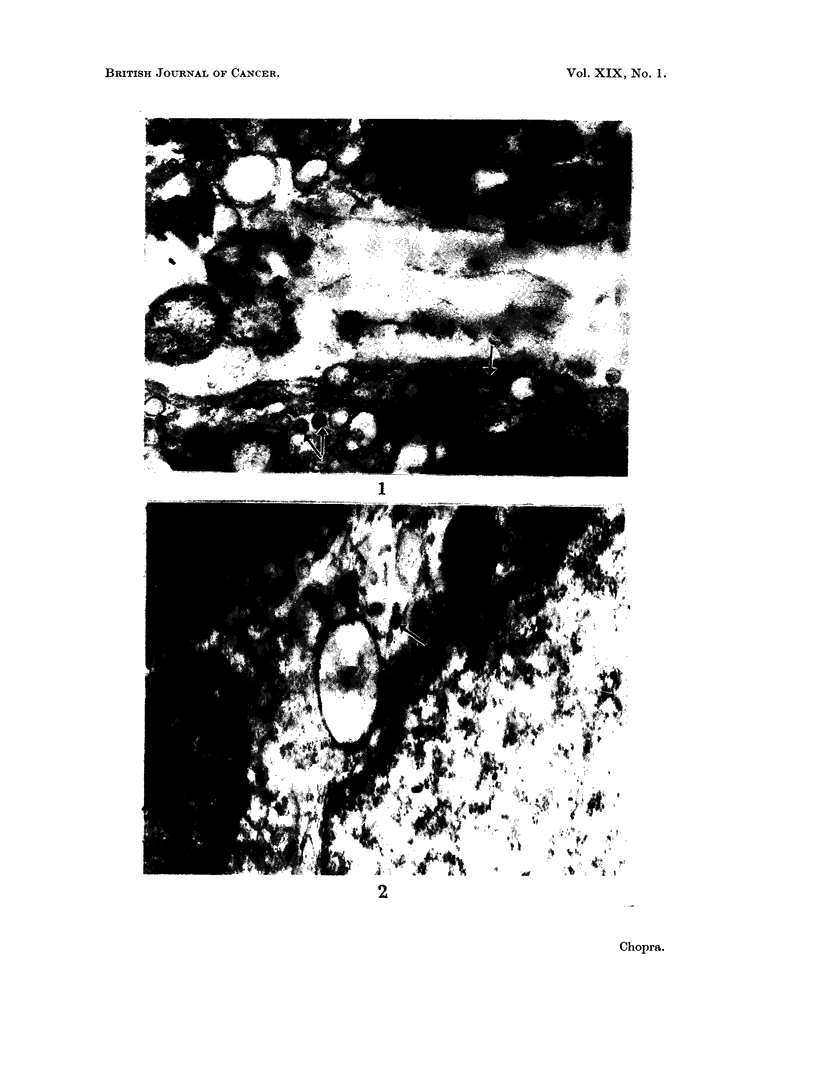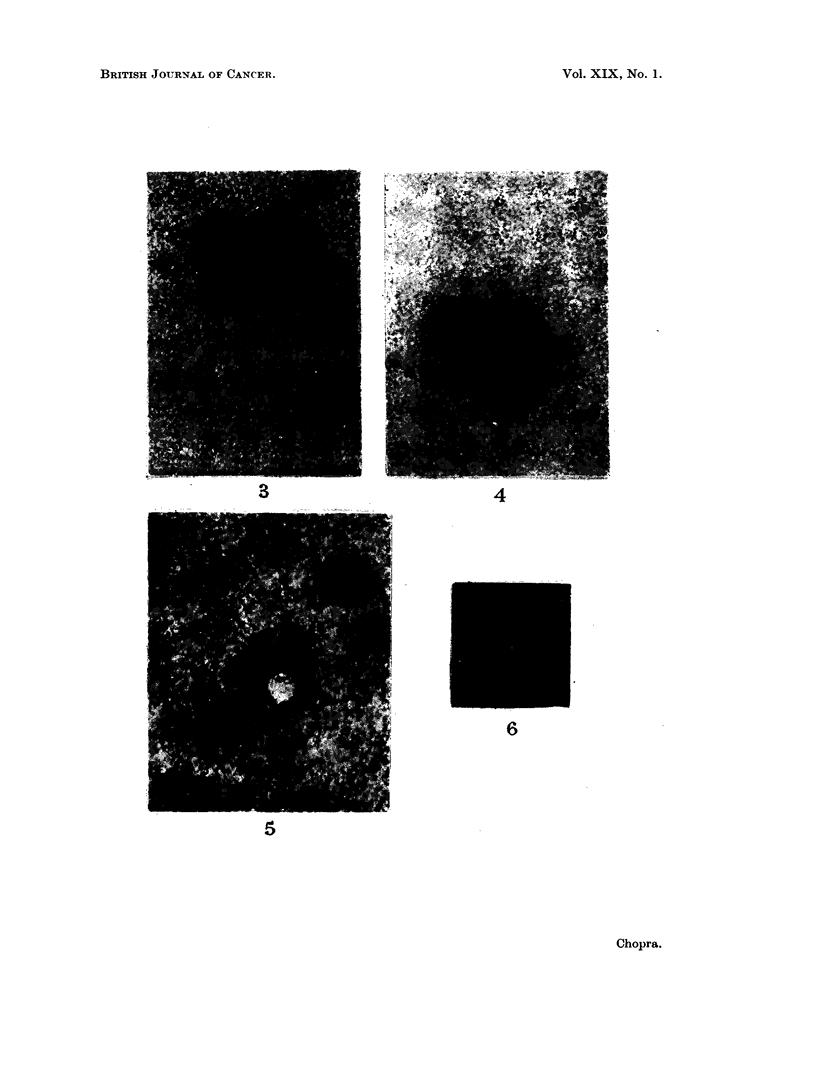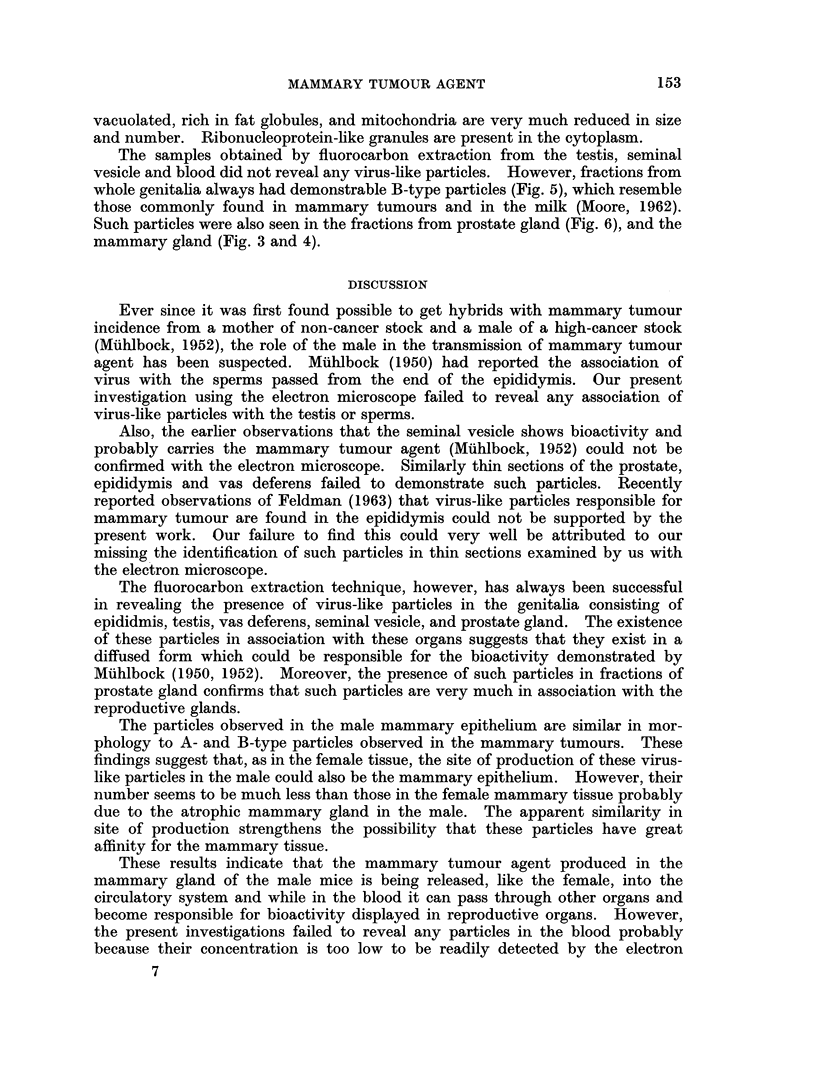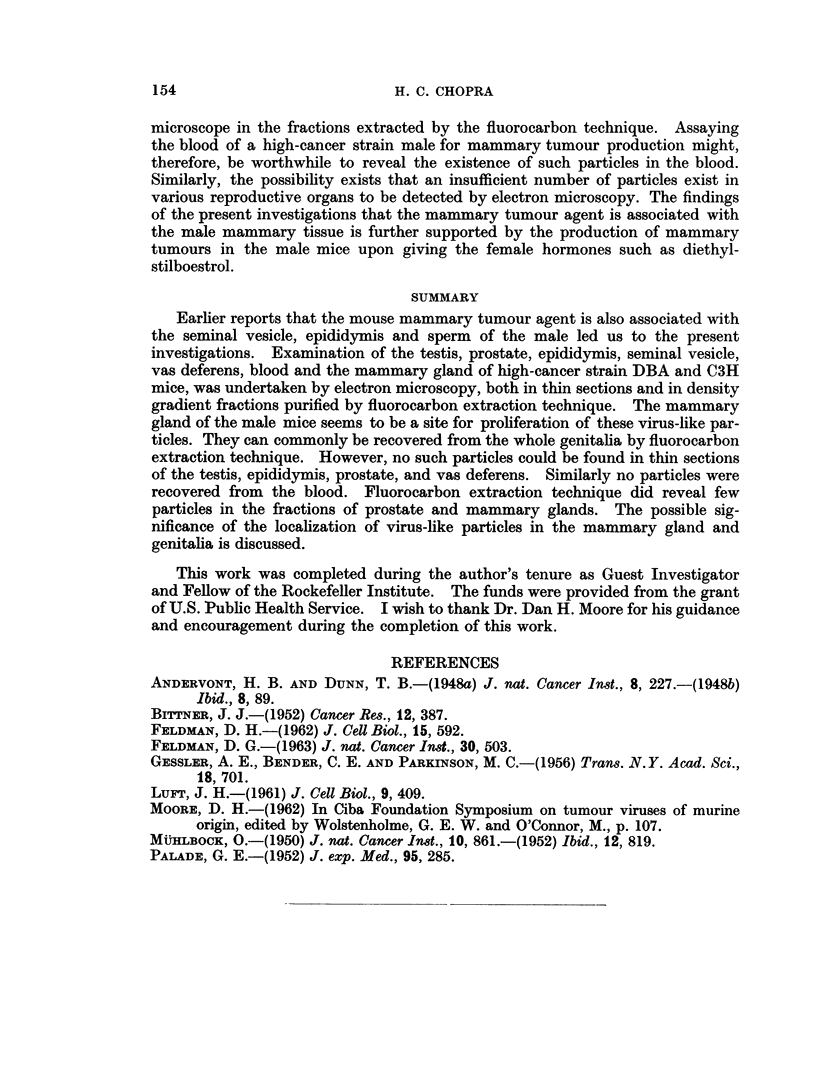# Study of Virus-Like Particles in Male Mice Carrying Mammary Tumour Agent

**DOI:** 10.1038/bjc.1965.17

**Published:** 1965-03

**Authors:** H. C. Chopra

## Abstract

**Images:**


### STUDY OF VIRUS-LIKE PARTICLES IN MALE MICE CARRYING MAMMARY TUMOUR AGENT

H. C. CHOPRA*

*From The Rockefeller Institute, New York, U.S.A.*

Received for publication October 28, 1964

SEVERAL investigations had been conducted in order to study the origin and distribution of mammary tumour agent (MTA) in various organs of high cancer strain mice. It (MTA) is known to be harboured also by the males of the same strains in their reproductive organs and accessory glands (Andervont and Dunn, 1948*a*) from which it can be transmitted to virus susceptible females in the course of repeated mating to these same females and eventually to their young ones (Andervont and Dunn, 1948*b*) through the milk (Bittner, 1952). Mühlbock (1950) established the presence of this agent in the sperms passed from the end of the epididymis. He (Mühlbock, 1952) had experimentally shown that agent free females develop mammary tumours after mating with the males of high-cancer strain. He had also shown that the seminal vesicle of such males shows biological activity for these agents and can produce mammary tumours in the females.

Recently Feldman (1963) has shown virus-like particles within the endoplasmic reticulum of the epididymis of both high-cancer strain, and agent free males.

Further investigations in this line of work were thought to be quite pertinent. It is observed that the atrophic male mammary gland of high-cancer strain mice is probably the site for proliferation of these virus-like particles which are stored in the genitalia for transmission to the females.

### METHODS

Virus-like particles were searched for in density gradient fractions purified by fluorocarbon extraction technique (Gessler, Bender and Parkinson, 1956) as well as in thin sections of various tissues by electron microscopy.

### *Fluorocarbon extraction technique*

The particles studied by dispersion technique were obtained by process of fluorocarbon extraction and differential centrifugation technique. The tissue was homogenized with a VirTis homogenizer in equal amounts of Freon and phosphate buffer, and centrifuged at 1500 r.p.m. for 10 minutes. The supernatant was further spun at 9000 r.p.m. and the pellet was discarded. Finally, the supernatant was ultracentrifuged at 39,000 r.p.m. for 1 hour and the pellet obtained was resuspended in 1 ml. buffered saline. This solution was layered over buffered rubidium chloride solution and centrifuged at 39,000 r.p.m. in the swinging bucket rotor for 22 hours. The band lying at the middle of the tube was isolated and dialysed with saline solution. The whole fractionation process was carried out in the cold (0–5° C.).

* Present address : Post-Graduate Institute for Medical Education and Research, Chandigarh, India.

*Electron Microscopy:*

(a) *Tissue sectioning.*—Tissue samples of testis, seminal vesicle, prostate, epididymis, vas deferens, and mammary gland from the known high-cancer strain male mice (DBA and C3H) were excised and immediately fixed on ice in Palade's (1952) buffered 1% osmium tetroxide for one hour. They were then rinsed in 50% alcohol; dehydrated in successive changes of 70%, 95%, and 100% ethanol. After thorough dehydration the material was put in propylene oxide for about 3 hours. These specimens were embedded in fresh Epon 812 (Luft, 1961) and allowed to polymerize in a 60° C. oven for 24 hours. Sections of the blocks were cut with glass knives on a Porter-Blum (prototype fabricated) thermal advance microtome. The sections were picked up on bare grids and stained in 1% lead hydroxide (Feldman, 1962) for 10–15 minutes, and the electron micrographs were taken in an RCA EMU–3F electron microscope.

(b) *Negative staining.*—A drop of the various fractions obtained by extraction technique were put on the grids coated with formvar and then a drop of 2% phosphotungstic acid (PTA) was immediately placed on these grids. After half a minute the drop was touched with torn filter paper to remove excess stain. The grids were examined after drying in an RCA EMU–3F electron microscope.

### RESULTS

Thin sections from various organs, namely, testis, epididymis, seminal vesicle, prostate gland and vas deferens obtained from high-cancer strain male mice failed to reveal any particles which could be correlated with the virus-like particles of breast cancer in mice. However, it could be observed in the thin sections of the male mammary tissue that virus-like particles were being budded off, and that these particles morphologically resemble A-type particles often seen in the cytoplasm of mammary tumours. These particles consist of a central core which is enclosed in a dense shell having a granular surface (Fig. 1).

Similarly a few particles which could be suspected as immature B-type particles are also observed in the mammary tissue (Fig. 2). Unlike the mature B-type particles, which are considered to be real mammary tumour agent, these particles lack dense nucleoid but instead contain material of light density enclosed in a membranous coat. Such particles are very rare in male mammary tissue probably due to the fact that the mammary gland in the male is very atrophic.

The mammary epithelial cells are very scanty and surrounded by the collagen fibres. The nucleus of such a cell is hyperchromatic and the cytoplasm is highly

### EXPLANATION OF PLATES

FIG. 1.—Section of male mammary gland from DBA mouse, showing A-type like particles (arrows) being budded off. × 20,000.

FIG. 2.—Section of mammary epithelium from male DBA mouse, showing particle suspected to be immature B-type without nucleoid. × 20,000.

FIG. 3.—Photomicrograph of negatively stained B-type particle separated from mammary gland of male C3H mouse. ×95,000.

FIG. 4.—Negatively stained B particle separated from the male mammary gland of male DBA mouse. × 95,000.

FIG. 5.—Negatively stained B particle separated from genitalial of male C3H mouse. ×50,000.

FIG. 6.—Negatively stained B particle separated from the prostate gland of DBA mouse. ×62,000.

1

2

Chopra.

3

4

5

6

Chopra.

vacuolated, rich in fat globules, and mitochondria are very much reduced in size and number. Ribonucleoprotein-like granules are present in the cytoplasm.

The samples obtained by fluorocarbon extraction from the testis, seminal vesicle and blood did not reveal any virus-like particles. However, fractions from whole genitalia always had demonstrable B-type particles (Fig. 5), which resemble those commonly found in mammary tumours and in the milk (Moore, 1962). Such particles were also seen in the fractions from prostate gland (Fig. 6), and the mammary gland (Fig. 3 and 4).

### DISCUSSION

Ever since it was first found possible to get hybrids with mammary tumour incidence from a mother of non-cancer stock and a male of a high-cancer stock (Mühlbock, 1952), the role of the male in the transmission of mammary tumour agent has been suspected. Mühlbock (1950) had reported the association of virus with the sperms passed from the end of the epididymis. Our present investigation using the electron microscope failed to reveal any association of virus-like particles with the testis or sperms.

Also, the earlier observations that the seminal vesicle shows bioactivity and probably carries the mammary tumour agent (Mühlbock, 1952) could not be confirmed with the electron microscope. Similarly thin sections of the prostate, epididymis and vas deferens failed to demonstrate such particles. Recently reported observations of Feldman (1963) that virus-like particles responsible for mammary tumour are found in the epididymis could not be supported by the present work. Our failure to find this could very well be attributed to our missing the identification of such particles in thin sections examined by us with the electron microscope.

The fluorocarbon extraction technique, however, has always been successful in revealing the presence of virus-like particles in the genitalia consisting of epididmis, testis, vas deferens, seminal vesicle, and prostate gland. The existence of these particles in association with these organs suggests that they exist in a diffused form which could be responsible for the bioactivity demonstrated by Mühlbock (1950, 1952). Moreover, the presence of such particles in fractions of prostate gland confirms that such particles are very much in association with the reproductive glands.

The particles observed in the male mammary epithelium are similar in morphology to A- and B-type particles observed in the mammary tumours. These findings suggest that, as in the female tissue, the site of production of these virus-like particles in the male could also be the mammary epithelium. However, their number seems to be much less than those in the female mammary tissue probably due to the atrophic mammary gland in the male. The apparent similarity in site of production strengthens the possibility that these particles have great affinity for the mammary tissue.

These results indicate that the mammary tumour agent produced in the mammary gland of the male mice is being released, like the female, into the circulatory system and while in the blood it can pass through other organs and become responsible for bioactivity displayed in reproductive organs. However, the present investigations failed to reveal any particles in the blood probably because their concentration is too low to be readily detected by the electron microscope in the fractions extracted by the fluorocarbon technique. Assaying the blood of a high-cancer strain male for mammary tumour production might, therefore, be worthwhile to reveal the existence of such particles in the blood. Similarly, the possibility exists that an insufficient number of particles exist in various reproductive organs to be detected by electron microscopy. The findings of the present investigations that the mammary tumour agent is associated with the male mammary tissue is further supported by the production of mammary tumours in the male mice upon giving the female hormones such as diethylstilboestrol.

SUMMARY

Earlier reports that the mouse mammary tumour agent is also associated with the seminal vesicle, epididymis and sperm of the male led us to the present investigations. Examination of the testis, prostate, epididymis, seminal vesicle, vas deferens, blood and the mammary gland of high-cancer strain DBA and C3H mice, was undertaken by electron microscopy, both in thin sections and in density gradient fractions purified by fluorocarbon extraction technique. The mammary gland of the male mice seems to be a site for proliferation of these virus-like particles. They can commonly be recovered from the whole genitalia by fluorocarbon extraction technique. However, no such particles could be found in thin sections of the testis, epididymis, prostate, and vas deferens. Similarly no particles were recovered from the blood. Fluorocarbon extraction technique did reveal few particles in the fractions of prostate and mammary glands. The possible significance of the localization of virus-like particles in the mammary gland and genitalia is discussed.

This work was completed during the author's tenure as Guest Investigator and Fellow of the Rockefeller Institute. The funds were provided from the grant of U.S. Public Health Service. I wish to thank Dr. Dan H. Moore for his guidance and encouragement during the completion of this work.

### REFERENCES

ANDERVONT, H. B. AND DUNN, T. B.—(1948*a*) *J. nat. Cancer Inst.*, **8**, 227.—(1948*b*) *Ibid.*, **8**, 89.
BITTNER, J. J.—(1952) *Cancer Res.*, **12**, 387.
FELDMAN, D. H.—(1962) *J. Cell Biol.*, **15**, 592.
FELDMAN, D. G.—(1963) *J. nat. Cancer Inst.*, **30**, 503.
GESSLER, A. E., BENDER, C. E. AND PARKINSON, M. C.—(1956) *Trans. N.Y. Acad. Sci.*, **18**, 701.
LUFT, J. H.—(1961) *J. Cell Biol.*, **9**, 409.
MOORE, D. H.—(1962) In Ciba Foundation Symposium on tumour viruses of murine origin, edited by Wolstenholme, G. E. W. and O'Connor, M., p. 107.
MÜHLBOCK, O.—(1950) *J. nat. Cancer Inst.*, **10**, 861.—(1952) *Ibid.*, **12**, 819.
PALADE, G. E.—(1952) *J. exp. Med.*, **95**, 285.